# Expression of the phagocytic receptors α_M_β_2_ and α_X_β_2_ is controlled by RIAM, VASP and Vinculin in neutrophil-differentiated HL-60 cells

**DOI:** 10.3389/fimmu.2022.951280

**Published:** 2022-09-27

**Authors:** Alvaro Torres-Gomez, Tara Fiyouzi, Claudia Guerra-Espinosa, Beatriz Cardeñes, Irene Clares, Víctor Toribio, Pedro A. Reche, Carlos Cabañas, Esther M. Lafuente

**Affiliations:** ^1^ Department of Immunology, Ophthalmology and Otorhinolaryngology, School of Medicine, Universidad Complutense de Madrid, Madrid, Spain; ^2^ Instituto de Investigación Sanitaria Hospital 12 de Octubre (i+12), Inflammatory Diseases and Immune Disorders (Lymphocyte Immunobiology Unit), Madrid, Spain; ^3^ Tissue and Organ Homeostasis Program (Cell-Cell Communication and Inflammation Unit), Centre for Molecular Biology "Severo Ochoa", Consejo Superior de Investigaciones Científicas (CSIC)-Universidad Autónoma de Madrid (UAM), Madrid, Spain

**Keywords:** phagocytosis, cytoskeleton, vasp, integrin expression, CR3 (CD11b/CD18), Vinculin, CR4 (CD11c/CD18), riam

## Abstract

Activation of the integrin phagocytic receptors CR3 (α_M_β_2_, CD11b/CD18) and CR4 (α_X_β_2_, CD11c/CD18) requires Rap1 activation and RIAM function. RIAM controls integrin activation by recruiting Talin to β_2_ subunits, enabling the Talin-Vinculin interaction, which in term bridges integrins to the actin-cytoskeleton. RIAM also recruits VASP to phagocytic cups and facilitates VASP phosphorylation and function promoting particle internalization. Using a CRISPR-Cas9 knockout approach, we have analyzed the requirement for RIAM, VASP and Vinculin expression in neutrophilic-HL-60 cells. All knockout cells displayed abolished phagocytosis that was accompanied by a significant and specific reduction in ITGAM (α_M_), ITGAX (α_X_) and ITGB2 (β_2_) mRNA, as revealed by RT-qPCR. RIAM, VASP and Vinculin KOs presented reduced cellular F-actin content that correlated with αM expression, as treatment with the actin filament polymerizing and stabilizing drug jasplakinolide, partially restored α_M_ expression. In general, the expression of α_X_ was less responsive to jasplakinolide treatment than α_M_, indicating that regulatory mechanisms independent of F-actin content may be involved. The Serum Response Factor (SRF) was investigated as the potential transcription factor controlling α_M_β_2_ expression, since its coactivator MRTF-A requires actin polymerization to induce transcription. Immunofluorescent MRTF-A localization in parental cells was primarily nuclear, while in knockouts it exhibited a diffuse cytoplasmic pattern. Localization of FHL-2 (SRF corepressor) was mainly sub-membranous in parental HL-60 cells, but in knockouts the localization was disperse in the cytoplasm and the nucleus, suggesting RIAM, VASP and Vinculin are required to maintain FHL-2 close to cytoplasmic membranes, reducing its nuclear localization and inhibiting its corepressor activity. Finally, reexpression of VASP in the VASP knockout resulted in a complete reversion of the phenotype, as knock-ins restored α_M_ expression. Taken together, our results suggest that RIAM, VASP and Vinculin, are necessary for the correct expression of α_M_β_2_ and α_X_β_2_ during neutrophilic differentiation in the human promyelocytic HL-60 cell line, and strongly point to an involvement of these proteins in the acquisition of a phagocytic phenotype.

## Introduction

The leukocytic integrins α_M_β_2_ (CD11b/CD18) and α_X_β_2_ (CD11c/CD18), also known as CR3 and CR4, respectively, are the main receptors of the complement fragment iC3b and their activation results in phagocytosis of iC3b-opsonized targets (1, 2). Both integrins can bind additional ligands such as fibrinogen (3), or intercellular adhesion molecule-1 (ICAM-1) on endothelial cells, participating in the phase of firm adhesion and fast migration during PMN extravasation to inflammation sites (4, 5).

Membrane expression of α_M_β_2_ and α_X_β_2_ greatly increases upon leukocyte activation. In unstimulated neutrophils, these integrins localize mainly in endomembranes (75% at gelatinase granules, 20% with secretory vesicles) and only a minor portion is expressed at the plasma membrane. Stimulation with chemoattracting agents (fMLP, LTB-4) and/or certain cytokines (IL-8, granulocyte-macrophage colony stimulating factor, platelet-activating factor, TNF-α) induces a rapid translocation of α_M_β_2_ and α_X_β_2_ to the plasma membrane, increasing its expression by 6 to 7-fold without apparently involving transcriptional regulation (6). α_M_β_2_ and α_X_β_2_ require a conformational change in order to be fully active and capable of binding their ligands with high affinity. This high affinity state can be induced by signaling pathways initiated from other receptors (pattern recognition receptors, chemokine receptors, anaphylatoxin receptor, among others) (7). These inside-out pathways converge in a central node, represented by the active form of the small GTPase Rap1 (Rap1-GTP) which interacts with its effector protein RIAM, promoting its translocation to the plasma membrane (8, 9). RIAM itself interacts with Talin releasing its auto-inhibition and triggering its recruitment to the plasma membrane, where Talin may bind to the proximal NPxY motif present in the cytoplasmic tail of the integrin β_2_ subunit (10, 11). This interaction results in α_M_β_2_ activation, which acquires the “open headpiece” conformation that allows for high-affinity ligand interaction (12). Upon ligand binding, α_M_β_2_ triggers outside-in signals that recruit protein complexes consisting of adaptor molecules, kinases, phosphatases, and actin polymerases, that regulate cell adhesion and phagocytosis, leading to crucial phenotypical and functional changes in myeloid leukocytes (13). Talin, RIAM, VASP and Vinculin have been identified as being key components of the Integrin Adhesion Complex (IAC), a subset of integrin proximal proteins of the integrin adhesome (14, 15). Signaling stemming from this complex determines cell adhesion and migration, but also other fundamental processes such as cell growth and differentiation (16).

We have previously reported that the adaptor molecule RIAM and its interacting partner VASP participate in this outside-in signaling pathway during complement-mediated phagocytosis (17). By interfering with RIAM expression in neutrophil-like HL-60 cells (RIAM KD) we have demonstrated that VASP recruitment at phagocytic cups is impaired and so is VASP phosphorylation at Ser^157^, which has been associated to its actin polymerization activity. In line with this observation, RIAM KD cells presented reduced F-actin content at phagocytic cups during complement-dependent phagocytosis. Knocking out VASP in HL-60 cells using CRISPR/Cas9 technology also resulted in a drastic decrease in phagocytic capacity with significant decrease in particle association. We concluded that RIAM worked as a relay for integrin complement receptors in outside-in signaling, coordinating integrin activation and cytoskeletal rearrangements *via* its interaction with VASP (17).

The importance of RIAM and Talin in integrin activation is highlighted by the effect of their deletion, where knockout mice display a phenotype reminiscent of Leukocyte Adhesion Deficiency (LAD) syndromes, with a pronounced neutrophilic (4-fold increase) and monocytic leukocytosis for RIAM^-/-^ mice that is even more pronounced for Talin1^-/-^ mice (>30-fold increase). LAD syndromes are characterized by recurrent infections due to ineffective neutrophil and macrophage function, and disrupted myeloid cell differentiation with extreme neutrophilia (~5 to 10-fold increases), caused by the egress of immature precursors (18–20). In the case of LAD-I patients, leukocyte expression of integrin β_2_ is greatly diminished, or absent, with a concomitantly reduced or null expression of its binding partner subunits α_L_, α_M_ and α_X_ (21).

Since RIAM^-/-^ had shown LAD-like defects, and we had observed an abolishment of phagocytosis when deleting its binding partner VASP, in this work we decided to dissect the effects of RIAM, VASP or Vinculin knockouts on α_M_β_2_ and α_X_β_2_ function and expression. By using CRISPR/Cas9 technology, we have generated RIAM^-/-^, VASP^-/-^ and Vinculin^-/-^ HL-60 cells. All these cell lines presented a common phenotype, a reduced expression of *ITGAM* (α_M_), *ITGAX* (α_X_) and *ITGB2* (β_2_) mRNA with their correspondingly low level of surface protein expression, which was not due to transport deficiencies. This defect in integrin subunit expression was accompanied by a reduction in cellular F-actin. Treatment with the actin-stabilizing drug jasplakinolide, which induces actin polymerization, alleviated the defect in integrin expression. Similarly, integrin subunit expression could be renormalized by reexpression of VASP in HL-60 VASP^-/-^ cells (HL-60 VASP KIs). Nuclear translocation and activation of the transcriptional co-activator MRTF-A, a cofactor to the Serum Response transcription Factor (SRF), is dependent on actin polymerization and was therefore a prime candidate to explain the observed phenotype. Analysis of MRTF-A subcellular distribution revealed differences in nuclear localization in comparison to parental HL-60 cells that could account for the defect in α_M_ and α_X_ expression. The reverse pattern was observed for the SRF co-repressor FHL-2, an antagonist for MRTF-A. We conclude that the expression of α_M_β_2_ and α_X_β_2_ integrins is dependent on the activity of RIAM, VASP and Vinculin, all of which have been previously shown to form complexes with each other. Since α_M_β_2_ and α_X_β_2_ are both markers for neutrophilic differentiation, a correct regulation of their expression could have implications in leukemic change and development during neutrophil differentiation.

## Material and methods

### Cell cultures

Human promyelocytic HL-60 (ATCC: CCL-240) and derived cell lines were cultured in 10 ml RPMI 1640 medium with 10% (v/v) fetal-calf serum (FCS), 1% (v/v) glutamine and 1% (w/v) penicillin-streptomycin (Lonza) (11, 17, 22) using Nunc™ 100 mm dishes (Thermo Scientific). Cells were differentiated into neutrophil-like HL-60 using 1 μM retinoic acid (Sigma) during at least 2 days. HEK 293T cells (ATCC: CRL-3216) were cultured in the same media in Nunc™ 100 mm dishes (Thermo Scientific) for adherent cells.

### Phagocytosis assays

Phagocytosis assays were carried out as previously described (11, 17). Briefly, fresh sheep red blood cells (RBCs) (Thermo Scientific) labelled with 2 μM DDAO-AM (Invitrogen) were incubated with sub-agglutinating concentrations of polyclonal rabbit IgM anti-sheep RBC cells (MyBioSource) and later treated with 10% C5-deficient human serum (Sigma) for complement opsonization. Differentiated HL-60 cells starved for 3 h in serum-free RPMI, were treated with 320 nM LPS (Sigma) for 30 minutes or 1 mM MnCl_2_ for 5 minutes. Cells were incubated with complement-opsonized RBCs (C3-RBC) or unopsonized RBCs as negative control, for 30 min at 37°C in a 1:10 ratio, and unbound RBCs were washed thrice with ice-cold PBS. To determine particle internalization, cell-bound RBCs were exposed to a 30 s hypotonic shock with distilled H_2_O, and isotonicity restored with an equal volume of twice-concentrated PBS. Cells were analyzed in a BD FACSCalibur II flow cytometer (BD Biosciences), using the FlowJo package (BD Biosciences) and expressed as Association Index (AI), indicating the number of cells with attached and engulfed particles, or Phagocytic Index (PI) indicating cells with internalized particles (11). These indexes are all normalized with respect to the AI for unstimulated control cells.

### Western blotting

Cell lysates were obtained from 10 million cells using GST Buffer (50 mM Tris-HCl pH 7.4, 100 mM NaCl, 2 mM MgCl_2_, 10% v/v glycerol, 1% v/v NP-40) supplemented with 1 mM PMSF, 25 mM NaF, 1 mM Na_3_VO_4_, and a protease inhibitor cocktail (Sigma). 50 μg protein were separated by SDS-PAGE electrotransferred to a nitrocellulose membrane, blocked and incubated with the following anti-human primary antibodies: rabbit IgG anti-VASP, (Cell Signaling), mouse IgG anti-α-Tubulin, (Sigma), mouse IgG anti-Vinculin (H-10 clone, Santa Cruz), sheep IgG anti-RIAM (R&D Systems) and mouse IgG anti-Talin (8D4 clone, Sigma). After washes, membranes were incubated with a secondary IRDye^®^ IgG anti-rabbit, anti-goat/sheep or anti-mouse fluorescent antibodies (Li-Cor). All antibodies were used as per the manufacturer’s instructions. The signal was then measured in a Li-Cor Odyssey imaging system and quantified using the ImageStudio software (Li-Cor).

### Gene knockout and gene transfection

Protein knockout lines were obtained using a CRISPR-Cas9 system and a double nickase strategy. Pairs of sgRNAs were designed using the Optimized CRISPR Design tool (23), and the highest scoring pairs were selected. To ensure the truncated proteins were non-functional, sgRNAs were directed towards the first common exon for all isoforms of VASP (exon 2), RIAM (exon 3) and Vinculin (exon 3).The corresponding pairs of sgRNAs for *VASP* (5’-CACCGGTAGATCTGGACGCGGCTGA-3’ and 5’-CACCGGCCAATTCCTTTCGCGTCGT-3’), *APBB1IP* (RIAM), (5’-CACCGATTTGTTCCATAACCAAGAG-3’ and 5’-CACCGCACTGGTATCAGCCAATATG-3’) *VCL*, (5’-CACCGTCAATTAGATAATCTCGAGC-3’ and 5’-CACCGGGGTCAAGGGGCATCCTCTC-3’) and their complementary oligonucleotide chains were ordered (Sigma), and cloned into BbsI-digested PX458 plasmid (23). Cell transfection was carried out using the Neon Transfection System (ThermoFisher) following manufacture instructions. For each nucleofection, 250 000 cells and mixture of 3 μg of the two sgRNA plasmids were employed. Cells were then transfected in a 10 μl volume using a single 35 ms and 1350 V pulse and left to recover for 24 h in RPMI 1640 10% FCS media without antibiotics. Cells were then sorted according to transient EGFP fluorescence using a FACS Aria Fusion cell sorter (BD Biosciences). EGFP-positive cells were diluted and cloned into p96 wells. Protein expression was then assessed through western blot and negative clones were selected. Sanger sequencing was used to analyze DNA editing in the selected clones, and sequences were compared to their respective genomic sequences. RIAM KO clone H9D2 presented 2 nt deletion (650 Del and 689 Del), VASP KO clone F6, presented a 46 nt deletion (394_439 Del), VASP KO clone F10 presented a 44 nt deletion (397_441 Del), Vcl KO clone A3 presented a 38 nt deletion (400_438 Del) and Vcl KO clone C4 presented a 35 nt deletion (405_440 Del). Once the transient EGFP expression was lost, we used VASP KO clones to generate the VASP rescue polyclonal cell lines F6 KI and F10 KI. This was done through retroviral transduction using the plasmid pMSCV-EGFP-VASP and HEK 293T packaging cells following a previously described protocol (17). Characterization of these cells was done *via* western blot.

### Integrin expression analysis and jasplakinolide treatment

Integrin expression was monitored by flow cytometry and Geometric Mean Fluorescence Intensity (GMFI) was determined and normalized against the GMFI obtained for isotype controls. Staining was performed in a p96 U-bottom plate (Thermo Scientific) Cells were fixed with 2% formaldehyde for 10 minutes and blocked for 30 minutes with a PBS buffer containing 1% BSA and 100 μg/ml human gamma globulin (Sigma). When required, the cells were permeabilized with 0.1% Triton X-100 in PBS buffer for 10 minutes before blocking. The following hybridoma-derived monoclonal mouse antibodies were used to detect protein expression: BEAR-1 (integrin α_M_) (24), Ts1/11 (integrin α_L_) (25), HC1/1 (integrin α_X_) (26), BU15 mAb anti-CD11c (Immunotools), Ts2/16 (integrin β_1_) (27), Lia2/3 (integrin β_2_) (28), PAINS-10 (tetraspanin CD9) (29), Vj1/12 (CD59) (30) and 5A6 (tetraspanin CD81) (31). Cells were washed and stained with a donkey anti-mouse Alexa Fluor^®^ 488 antibody (Life Technologies), as per the manufacturer’s instructions. To determine total cellular F-actin, Phalloidin-Alexa Fluor^®^ 647 (Life Technologies) was used. When indicated, cells were treated with 1 µM jasplakinolide (Santa Cruz) 24 hours previous to completing the retinoic acid-induced differentiation.

### Integrin transcript analysis through RT-qPCR

Quantification of mRNA levels were performed using the RT-qPCR service offered by the Genomics unit of the Parque Científico de Madrid, Madrid, Spain. Briefly, 6x10^6^ retinoic acid differentiated HL-60 cells per assay were used. Quantification was performed with both triplicate biological replicates and technical replicates. mRNA was extracted using a miRNAEasy kit (Thermo Scientific). cDNA and RT-minus samples were prepared as per the service’s standardized protocols. PCR primers and TaqMan probes were acquired from Applied Biosystems (Thermo Fisher) for the following genes: *ITGAM* (α_M_ integrin subunit), *ITGAX* (α_X_ integrin subunit), *ITGAL* (α_L_ integrin subunit), *ITGB2* (β_2_ integrin subunit), *APBB1IP* (RIAM), and *GAPDH* and 18S rRNA were used as reference genes. Since calculated efficiencies for amplification of the gene of interest and reference genes were similar and close to 100%, fold change in mRNA expression was calculated using the Livak-Schmittgen ΔΔCT method (32).

### Fluorescence microscopy

Differentiated HL-60 cells were seeded on PLL (Poly-L-Lysine) coated glass slides, then treated with 1 µM jasplakinolide (Santa Cruz) for 2 hours or left unstimulated, and fixed with 4% paraformaldehyde for 10 minutes. Cells were permeabilized with PBS 0.3% Triton X-100 for 10 minutes and incubated with the indicated primary antibodies: mouse anti-MRTF-A (G8 clone, SantaCruz), rabbit anti-FHL-2 (Abcam) or biotin-labelled mouse Ts2/16 (prepared in house using a Biotin labelling kit purchased from Sigma, as described elsewhere (29). Primary antibodies were used as per the manufacturer’s instructions (1:20 anti-MRTF-A, and 1:100 anti-FHL-2), or 10 µg/ml of biotin-labelled purified antibody. Cells were stained with secondary antibodies (donkey anti-rabbit or donkey anti-mouse Alexa Fluor^®^ 647 conjugated antibodies, Life Technologies) and 488-conjugated streptavidin (Life Technologies). All antibodies were used as per the manufacturer’s instructions, in the presence of excess human gamma globulin (100 μg/ml) and 1% BSA as blocking agents. Imaging was performed using an LSM710 confocal laser scanning microscope coupled to an AxioImager M2 microscope (Zeiss) and analyzed using the ImageJ software package.

### Statistical analysis

Figures were prepared to show either representative results or mean ± standard deviation (SD) of at least 3 independent experiments (repetitions are stated in the figure legends). Significance between means was determined using a multiple comparisons ANOVA followed by a *post-hoc* Dunnett’s test to identify differences between groups. P-values less than 0.05 were considered statistically significant. To signal the degree of significance, asterisks were used as follows: a single asterisk denotes a significance of p<0.05; a double asterisk, p<0.01, a triple asterisk p<0.005 and a quadruple asterisk, p<0.0001. Finally, ns is used to denote no significance. Statistical calculations, data handling and graphing were performed on Microsoft Excel 2016 (Microsoft, Redmond, WA, USA) and GraphPad Prism 6 (GraphPad Software, San Diego, CA, USA).

## Results

### RIAM, VASP and Vinculin knockouts abolished phagocytosis

We previously determined that correct VASP expression is necessary for particle engulfment during phagocytosis, since VASP knockout resulted in an abolished phagocytosis and VASP overexpression significantly reduced phagocytic efficiency (17). Here, we further analyze the requirement for expression of VASP and VASP-interacting proteins, namely RIAM and Vinculin, in the process of complement-mediated phagocytosis.

**Figure 1:**
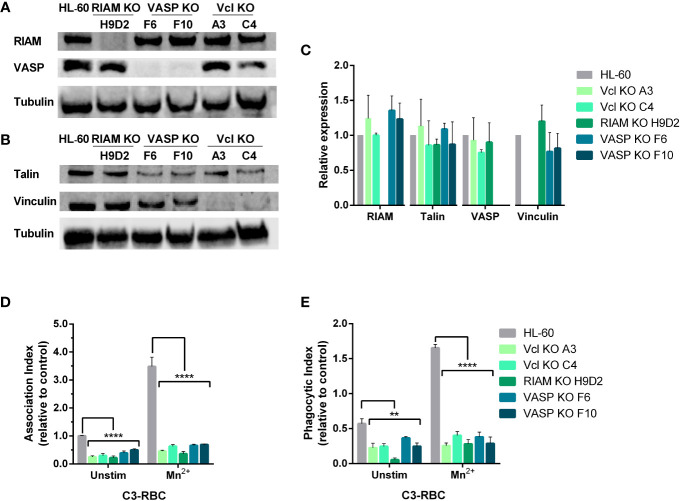
Knockout of either RIAM, VASP or Vinculin, abolishes phagocytosis. **(A, B)** Newly generated HL-60 Vinculin (Vcl), RIAM and VASP knockout monoclonal cell lines were tested for integrin related protein expression by western blot. **(C)** Quantification of protein expression in neutrophil-like HL-60 cells and derived knockouts analyzed by western blot. Results are represented as relative to HL-60 levels and are from 5 independent experiments. **(D, E)** Phagocytic cells were challenged with complement opsonized sheep red blood cells (C3-RBC) after being stimulated with 1 mM MnCl_2_ or left unstimulated, and Association (AI) and Phagocytic (PI) indexes were obtained. Data are normalized with respect to the AI of unstimulated C3-RBC-challenged HL-60 cells (control cells). Data are presented as mean ± SD, where the error bars denote standard deviation. Significance (ANOVA) has been calculated with respect to HL-60 controls, ** denotes p<0.01 and ****,p<0.0001.

Knockout of RIAM and Vinculin was performed using a double nick strategy and a transitory transfection using a CRISPR-Cas9 system. Western blot confirmed that all-*trans* retinoic acid differentiated HL-60 knockouts did not express detectable levels of the knocked-out proteins and demonstrated that genetic deletion of one of the genes from IAC does not affect the expression of the rest of components assessed (**Figures 1A–C**).

Since our prior reports indicated that VASP knockout abolishes particle internalization (17), RIAM and Vinculin (Vcl) knockout clones were subjected to a phagocytosis assay (**Figures 1D, E**). We observed that similarly to the results obtained from VASP deficient cells, RIAM and Vinculin knockout clones presented a drastically diminished Association Index (AI) that was detected even at basal state, demonstrating a 70-75% for Vcl KOs, and an 80% reduction for RIAM KOs (**Figure 1D**). This diminished binding capacity in the absence of stimulation was suggestive of defects in the adhesive properties of these cells. When integrins were activated *via* outside-in signaling using MnCl_2_ (Mn^2+^), all knockouts showed defective particle association, as stimulation was barely able to increase AI values to that of unstimulated control HL-60 cells. This defect in activation seemed to be more pronounced in RIAM KOs, a result which is in line with previous reports using RIAM-specific shRNA in HL-60 cell lines (10, 11, 17).

With regards to particle internalization (**Figure 1E**), all knockouts showed severely affected PI values, where again RIAM KO stood out as the most affected, as phagocytosis was barely detectable in these conditions. Contrasting with parental HL-60 cells, most KOs were incapable of responding to Mn^2+^, and no significant change was observed between unstimulated and stimulated cells (1.7-fold increase in KOs cells versus the 3.5-fold increase in HL-60 parental cells). It is worth noting that the response detected in KO cells could result from limitations in determining PI in unstimulated conditions.

Overall, the effects observed in phagocytosis seem to indicate a profound alteration in integrin activation and in the cytoskeletal rearrangements necessary for particle internalization. RIAM and VASP are reported to have an impact on F-actin content (8, 11) and potentially modulate transcriptional activity. The absence of IAC components could also have an effect in integrin stability and integrin recycling. Moreover, we speculated that integrin expression could be also affected in knockout cells.

### Expression of α_M_β_2_ and α_X_β_2_ integrins is reduced in RIAM, VASP and Vinculin knockout cells

**Figure 2:**
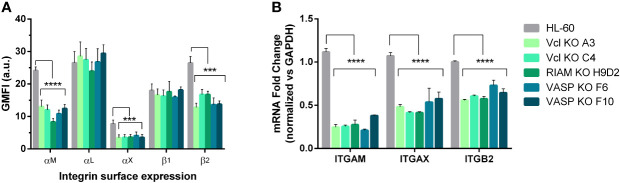
α_M_ and α_X_ expression is dependent on Vinculin, RIAM and VASP expression. **(A)** Vinculin (Vcl), RIAM and VASP knockout cell lines and HL-60 parental cells were differentiated into neutrophilic-like cells with 1 μM all-*trans* retinoic acid (RA) and stained with monoclonal antibodies specific for α_M_, α_L,_ α_X,_ β_1_, β_2_ integrin subunits. The geometric mean fluorescence intensity (GMFI) was obtained by flow cytometry and data represented as relative to HL-60 levels and are from 24 independent experiments done in duplicate. **(B)** Expression of *ITGAM* (α_M_), *ITGAX* (α_X_), and *ITGB2* (β_2_) mRNA levels was determined by RT-qPCR in neutrophil-like RIAM, VASP and Vcl HL-60 knockouts. Results are represented as relative to GAPDH mRNA and are from 3 independent experiments done in triplicate. Data are presented as mean ± SD, where the error bars denote standard deviation. Significance (ANOVA) has been calculated with respect to HL-60 controls, *** denotes p<0.005, and ****, p<0.0001.

To further characterize the phenotype observed in all knockouts, we analyzed the surface expression of α_M_, α_X_ and α_L_ subunits that form complement receptors CR3 and CR4 and integrin LFA1 respectively, together with β_2_ and β_1_ integrin subunits and other membrane integral proteins CD59, CD9 and CD81 as controls (**Figure 2A** and **Supplemental Figure 1B**). We observed that expression of the α_M_ subunit was significantly reduced (74-50%) as well as the expression of α_X_ which in some cases was virtually undetectable in KOs. The reduction in α_X_ and α_M_ expression was accompanied by a proportional reduction in β_2_ subunit expression in all KO clones (40-50%) (**Figure 2A**, **Supplemental Figure 1A**). No statistically significant differences were observed in the expression of α_L_ subunit, β_1_ integrin or tetraspanins CD9, and CD81 (all integrin related molecules) or CD59, a molecule unrelated to integrins (**Supplemental Figure 1B**). We confirmed that this reduction was also maintained when total cellular integrin expression was analyzed in permeabilized cells (**Supplemental Figure 1C**), thus ruling out a defect in protein transport to the plasma membrane. Since the observed reductions in α_M_ and α_X_ were similar in all clones, this suggests a similar mechanism may be involved in controlling expression of both CR3/α_M_β_2_ and CR4/α_X_β_2_ complement receptors, that requires the expression of RIAM, VASP and Vinculin. Furthermore, this mechanism appears to be highly specific for these integrin subunits, as it only affects the alpha subunits α_M_ and α_X_, whilst not affecting the closely related α_L_.

Next, we determined whether the decreased expression of α_M_ and α_X_ subunits was also detectable at mRNA level through RT-qPCR (**Figure 2B** **)**. After neutrophilic differentiation, all HL-60 knockouts cell lines showed a statistically significant 60-80% reduction in *ITGAM* (α_M_ gene) mRNA, and 40-50% for *ITGAX* (α_X_ gene) mRNA expression with respect to parental HL-60 cells, confirming a downregulated transcription. We also observed a decrease in *ITGB2* (β_2_ gene) mRNA levels that was comparable to the reduction observed in β_2_ expression at the plasma membrane. This downregulation was not observed for *ITGAL* (α_L_ gene) or *APBB1IP* (RIAM) in the VASP knockout (**Supplemental Figure 2A**). This indicates that the amount of β_2_ subunit produced in RIAM, Vcl and VASP KOs was enough to yield normal levels of integrin α_L_β_2_.

### Defective upregulation of integrins α_M_β_2_ and α_X_β_2_ during differentiation correlates with reduced F-actin content

**Figure 3:**
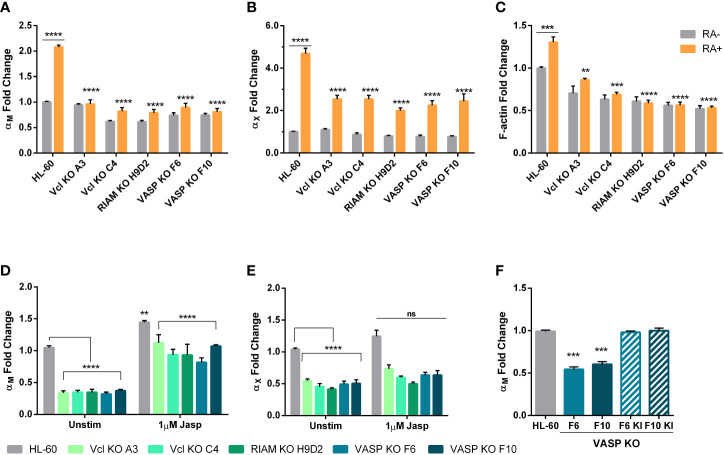
Upregulation of α_M_ and α_X_ expression during HL-60 neutrophilic differentiation depends on Vinculin, RIAM and VASP expression and is related cellular F- actin content. **(A, B)** Vinculin (Vcl), RIAM and VASP knockout cell lines and HL-60 parental cells were differentiated into neutrophilic-like cells with 1 μM all-*trans* retinoic acid treatment (RA+) or maintained undifferentiated (RA-), and expression of α_M_ and α_X_ integrins was analyzed by flow cytometry. **(C)** Cellular F-actin content was analyzed using fluorescently labeled phalloidin in HL-60 knockout cell lines and parental cells, in undifferentiated or differentiated cells. **(D, E)** Vinculin, RIAM and VASP knockout cell lines and HL-60 parental cells were treated with a 2 h 1 µM jasplakinolide stimulation, followed by a 24 h resting period during neutrophilic differentiation. Then, expression of α_M_ and α_X_ integrins was analyzed by flow cytometry. **(F)** Expression of α_M_ integrin was analyzed in VASP F6 and F10 knockout clones and in VASP knock-in polyclonal cell lines F6 KI and F10 KI. Results are represented as GMFI relative to HL-60 wild type levels and are from at least 3 independent experiments done in triplicate. Data are presented as mean ± SD, where the error bars denote standard deviation. Significance (ANOVA) has been calculated with respect to HL-60 controls, ** denotes p<0.01, *** p<0.005, and **** p<0.0001, and ns denotes no significance.

Next, we investigated whether the reduced expression of α_M_ and α_X_ in RIAM, VASP and Vinculin KO cells was caused by a failure to induce its expression during HL-60 differentiation. In HL-60 cells, treatment with retinoic acid induced a two-fold increase in α_M_ and around a five-fold increase in α_X_ expression (**Figure 3**), as well as an increase in RIAM and VASP expression (**Supplemental Figures 2B, C**). However, in the knockout clones, treatment with RA only induced a small increase in α_M_ expression reaching a level comparable to undifferentiated wild-type cells (**Figure 3A**), and a modest 2.5-fold increase in α_X_ expression compared to parental HL-60 cells (**Figure 3B**). This is suggestive of an impairment in the transcriptional activation of these integrins that takes place during neutrophilic differentiation, and is more acute in the case of α_M._

Prior studies revealed that RIAM silencing resulted in a reduction in F-actin content (8). It is also well known that the G:F actin ratio determines the activation of transcriptional regulation programs. Hence, we analyzed the total cellular F-actin content in RIAM, VASP and Vinculin KO cells (**Figure 3C**). In all cases F-actin content was diminished, with RIAM and VASP knockout clones showing a strong reduction (40-50%). This is in agreement with the previously observed defects in phagocytic capacity and prior reports linking RIAM and VASP with the control of cytoskeletal rearrangements necessary for particle engulfment (11, 17). F-actin content was also reduced in Vinculin knockouts but more moderately (30-37%). Differentiation induced a modest yet significant increase (31%) of total F-actin content in wild type HL-60 cells, but not in RIAM VASP or Vinculin knockout cells, which were unresponsive to retinoic acid. Overall, this reduction in total F-actin content, correlated with the observed defect in α_M_ expression.

Since F-actin content is capable of controlling gene expression, and it seemed to be the thread connecting all three knockouts, we tried to reverse the phenotype using the actin stabilizer jasplakinolide (Jasp), which induces actin polymerization. Indeed, this treatment induced a significant 3-fold increase in α_M_ levels for all knockouts, causing them to reach levels comparable to those of unstimulated parental cells, while having a modest effect on control HL-60 cells (1.4-fold increase) (**Figure 3D**). However, the effect of jasplakinolide in reverting α_M_ expression was partial, as knockouts could not reach the expression levels induced by this drug in parental cells. Nonetheless, this served as a proof-of-concept that F-actin levels are capable of controlling α_M_ expression in neutrophils. For α_X_ jasplakinolide treatment only induced a marginal 1.2-fold increase in expression, indicating a less relevant implication of cellular F-actin content in the control of α_X_ expression (**Figure 3E**).

Finally, to ensure that the defect in α_M_ expression was due to the gene deletion of the studied proteins, VASP was knocked-in in the two knockout clones, yielding the lines VASP F6 KI and F10 KI (**Supplemental Figure 2D**). When α_M_ levels were assessed (**Figure 3F**), these cells were indistinguishable from parental HL-60 cells. This served as a demonstration that genetic reconstitution of VASP was able to revert the observed phenotype and that therefore the observed effect was not due to the experimental system used.

### Distribution of the SRF co-regulators MRTF-A and FHL-2 is controlled by RIAM, VASP and Vinculin expression

The Serum Response Factor (SRF) transcription factor along with its co-activator MRTF-A are described as the main targets of actin dynamics (33–35). MRTF-A is sequestered in an inactive state in the cytoplasm by binding monomeric G-actin (36). Actin filament elongation reduces total cellular G-actin and leads to the dissociation of MRTF-A-G-actin complexes, and therefore allows MRTF-A nuclear import and subsequent activation of SRF-mediated transcription.

**Figure 4:**
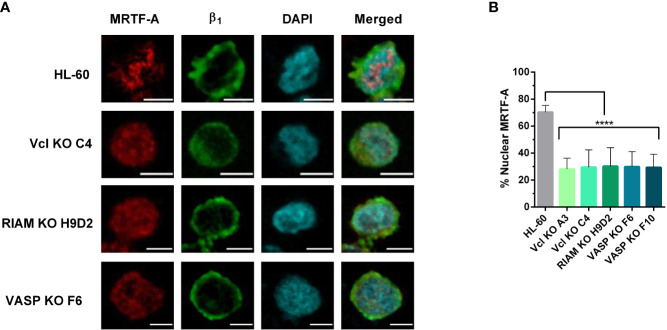
MRTF-A distribution is altered in Vinculin, RIAM and VASP knockouts. **(A)** HL-60 parental cells and the knockouts for Vinculin (Vcl), RIAM and VASP were differentiated into neutrophil-like cells, fixed, permeabilized and fluorescently labelled with an anti-MRTF-A mAb, DAPI for nuclear staining and anti-β_1_ integrin to delimit the plasma membrane. Images show representative results from 3 independent experiments analyzed by confocal fluorescence microscopy. Bars indicate 5 µm. **(B)** Quantification of MRFT-A nuclear distribution in these images is represented. Results are represented as relative to the wild-type nuclear ratio and are from 3 independent experiments with at least 50 cells. Data are presented as mean ± SD, where the error bars denote standard deviation. Significance (ANOVA) has been calculated with respect to HL-60 controls, **** denotes p<0.0001.

To test the hypothesis that the SRF pathway could be involved in regulating α_M_ integrin expression, we studied MRTF-A subcellular localization in wild type and knockout lines using fluorescent microcopy (**Figure 4A**). For wild type cells, MRTF-A showed a punctate stain pattern, which co-localized with DAPI. This pattern implies a nuclear translocation, and is suggestive of an active transcription of SRF-controlled genes. In contrast, knockouts presented a diffuse staining pattern with MRTF-A predominantly distributed in the cytoplasm.

The extent of MRTF-A nuclear translocation was determined by fluorescent signal quantification. (**Figure 4B**). For each cell we defined the total fluorescence (or integrated density in ImageJ) in the MRTF-A channel for regions delimited by the cortical β_1_ integrin staining channel, as total cellular MRTF-A, and the fluorescence which co-localized with the nuclear DAPI stain, as the nuclear MRTF-A. While HL-60 cells showed a primarily nuclear localization of MRTF-A (~74%) the three HL-60 knockouts showed little nuclear translocation (<30%), indicating that knockout of these proteins results in a statistically significant and drastic reduction in MRTF-A translocation. These results are in agreement with our previous observations that knockouts present reduced levels of F-actin and suggest that defective SRF activity plays a role in the loss of α_M_ expression observed for the knockouts.

**Figure 5:**
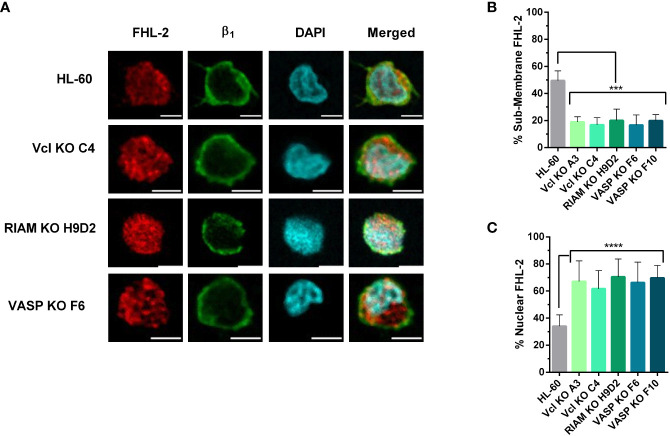
FHL-2 nuclear translocation is enhanced in Vinculin, RIAM and VASP knockouts. **(A)** HL-60 parental cells and the knockouts for Vcl, RIAM, VASP HL-60 cells were differentiated into neutrophilic-like cells with 1 μM all-*trans* retinoic acid for 48 h, fixed, permeabilized and fluorescently labelled using anti-FHL-2 and anti-β_1_ integrin antibodies and DAPI. Confocal microscopy images were analyzed using the ImageJ software package and are representative results from 3 independent experiments. Bars indicate 5 µm. **(B, C)** Subcellular distribution of FHL-2. The graphs represent the quantification of images. Results are represented as relative to the wild-type nuclear ratio and are from 3 independent experiments with at least 50 cells. Data are presented as mean ± SD, where the error bars denote standard deviation. Significance (ANOVA) has been calculated with respect to HL-60 controls, ***denotes p<0.005, and ****, p<0.0001.

Similarly, we studied the subcellular localization of the SRF corepressor FHL-2 (**Figure 5**). FHL-2 is known to compete with MRTF-A for SRF-binding and acts as a negative feedback loop, since MRTF-A is capable of inducing FHL-2 expression (37). Wild type cells showed FHL-2 enrichment in the cytoplasm in close proximity to β_1_ integrins, which is indicative of a membranous localization. However, for knockouts FHL-2 staining followed a cytoplasmic and nuclear distribution. The FHL-2 fluorescence was quantified (**Figures 5B, C**). Signals co-localizing with DAPI were assigned as nuclear FHL-2 and sub-membranous when localizing with integrin β_1_. Total cellular FHL-2 was also determined. Our data confirmed that for RIAM, VASP and Vinculin knockouts FHL-2 localization was mainly nuclear (~60%) with a minor proportion at the sub-membrane (~20%), while in HL-60 parental cells, FHL-2 was more abundant at the membrane (~50%) and only a 35% was nuclear. This result suggests that the expression of RIAM, VASP, and Vinculin may be required to retain FHL-2 close to the cytoplasmic membrane, preventing its corepressor activity and allowing proper integrin expression.

### Jasplakinolide treatment renormalizes SRF co-regulator subcellular localization

**Figure 6:**
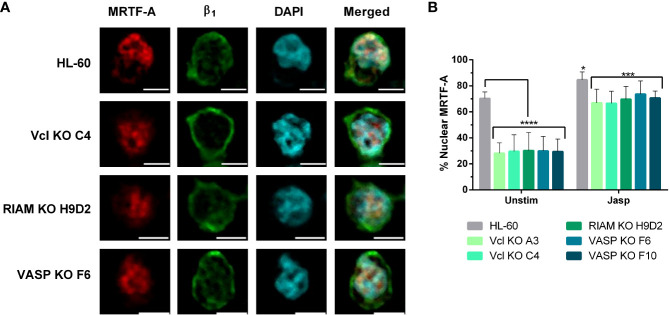
Jasplakinolide induces MRTF-A nuclear translocation in Vinculin, RIAM and VASP knockouts. **(A)** Differentiated HL-60 parental cells and HL-60 knockouts for Vcl, RIAM and VASP were adhered to slides, then subjected to a 1 µM 2 h jasplakinolide stimulation, fixed, permeabilized and fluorescently stained to determine MRTF-A localization. β_1_ integrin was used to delimit the plasma membrane and DAPI was used as a nuclear stain. Confocal microscopy images were analyzed using the ImageJ package and are representative results from 3 independent experiments. Bars indicate 5 µm. **(B)** Quantification images from the untreated cells shown on **Figure 4** and jasplakinolide-treated cells on **(A)**. Results are represented as relative to the wild-type nuclear ratio and are from 3 independent experiments with at least 50 cells. Data are presented as mean ± SD, where the error bars denote standard deviation. Significance (ANOVA) has been calculated with respect to HL-60 controls, * denotes p<0.05, ***,p<0.005, and ****,p<0.0001.

The data shown in **Figure 3** indicates that jasplakinolide treatment in RIAM, VASP, and Vinculin KO cell lines was able to increase α_M_ expression. Thereby, we assessed whether this treatment could also revert the subcellular localization of MRTF-A in knockout cells to resemble wild type cells (**Figure 6**). As expected, we observed that jasplakinolide treatment increases MRTF-A signal at the nucleus for HL-60 controls, since the MRTF-A closely co-localized with the nuclear DAPI stain. MRTF-A also displayed a clear nuclear localization in all knockout cells indicating that jasplakinolide treatment positively affected MRTF-A nuclear translocation, bypassing the lack of expression of the knocked-out proteins. Furthermore, for all jasplakinolide-treated cells, barely any cytoplasmic MRTF-A was observable.

Next, we analyzed the extent of MRFT-A translocation by fluorescent signal quantification in jasplakinolide treated cells (**Figure 6B**) and compared to untreated cells, following the approach used in **Figure 4B**. In jasplakinolide-treated cells we observed a statistically significant increase in nuclear localization for MRTF-A in all cells, compared to untreated cells. Such increase was minor in HL-60 cells (1.2-fold), as most MRTF-A was nuclear prior to the jasplakinolide treatment, but quite significant in knockout cells (2.3 and 2.5-fold). This fold change correlated with the increase in α_M_ expression for differentiated HL-60 cells observed in **Figure 3D** (2-fold), suggesting that the pathway F-actin-MRTF-A/SRF is critically involved in the expression of α_M_.

**Figure 7:**
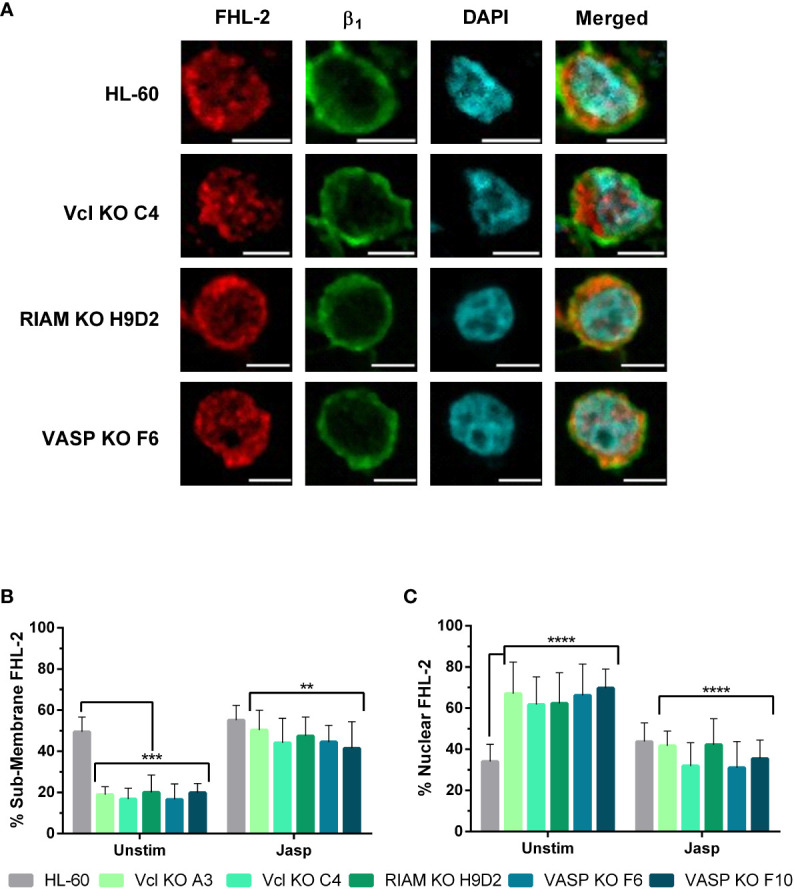
Jasplakinolide reduces nuclear localization of FHL-2 in Vinculin, RIAM and VASP knockouts. **(A)** Differentiated HL-60 parental cells and HL-60 knockouts for Vcl, RIAM and VASP were adhered to slides, then subjected to a 1 µ M 2 h jasplakinolide stimulation, fixed, permeabilized and fluorescently stained to determine FHL-2 localization. β_1_ integrin was used to delimit the plasma membrane and DAPI was used as a nuclear stain. Confocal microscopy images were analyzed using the ImageJ package and are representative results from 3 independent experiments. Bars indicate 5 μm. **(B, C)** Subcellular distribution of FHL-2. The graphs represent the quantification of images from untreated cells shown on **Figure 5** and jasplakinolide-treated cells shown on **(A)**. Results are represented as relative to the parental HL-60 membrane and nuclear ratio and are from 3 independent experiments with at least 50 cells. Data are presented as mean ± SD, where the error bars denote standard deviation. Significance (ANOVA) has been calculated with respect to HL-60 controls, ** denotes p<0.01, ***, p<0.005, and ****, p<0.0001.

FHL-2 subcellular localization was also assessed in jasplakinolide treated cells (**Figure 7**). In parental cells, FHL-2 showed a sub-membranous localization as judged by its close proximity to β_1_ integrin. FHL-2 was also present in the nucleus for jasplakinolide treated HL-60 cells, although to a lesser extent, coinciding with reports showing that MRTF-A signaling induces FHL-2 expression acting as a negative feedback loop (37). Although some nuclear distribution was also retained, knockouts displayed an observable FHL-2 enrichment at the cytoplasm and at the sub-membranous zone induced by jasplakinolide treatment (**Figure 7A**).

We analyzed the extent of FHL-2 translocation by fluorescent signal quantification in jasplakinolide treated cells (**Figures 7B, C**), following the same criteria used in **Figures 5B, C**. Jasplakinolide treatment induced an increase in FHL-2 membrane distribution (55%) on HL-60 parental cells compared to untreated cells, leading to the equal distribution of this protein between the membrane and nuclear compartments. For all knockouts jasplakinolide also increased FHL-2 localization at the membrane (from 20% to ~50%) and decreased nuclear FHL-2, compared to untreated cells. This renormalization of FHL-2 subcellular distribution correlates with our previous results showing a similar correction of MRTF-A nuclear translocation (**Figure 6**) and integrin α_M_ expression (**Figure 3C**) after jasplakinolide treatment.

## Discussion

Myeloid cell function is critically dependent on the correct expression of cell adhesion molecules as well as downstream effectors controlling their activation. Disruption of integrin expression or defects in proteins involved in their activation cause immunodeficiencies, as is the case of Leukocyte Adhesion Deficiencies (LAD), which are due to defects in β_2_ integrin or Kindlin-3 expression or function (18–20). The results presented here in HL-60 cells outline that the upregulation of α_M_ and α_X_ observed during myeloid differentiation depends on the correct expression of RIAM, VASP and Vinculin, which are proteins involved in actin cytoskeletal dynamics and integrin signaling.

We demonstrate that deletion of either RIAM, VASP or Vinculin causes a profound defect on the expression of CR3 and CR4 receptors without affecting other IAC components, in line with previous reports. RIAM knockout in mice had no detectable influence on Talin-1, Kindlin-3, Rap1 or Cal-DAG expression in platelets, macrophages, or PMNs (38) and Vinculin deletion in MEFs does not affect the expression of Talin, Paxillin, FAK, Zyxin, or VASP (39).

Similarly, it has been reported that IAC component-deficient cells frequently display defects on the expression of specific integrin subunits in a cell dependent context (39–43). Talin-1 deletion affects β_3_ expression, but not β_1_ in osteoclasts (44), while Talin-1 and Talin-2 double knockout exhibited increased β_3_, α_5_ and a slight increase in α_V_, but retained normal levels of β_1_ in fibroblasts (45). In B-cells, Talin-1 deletion caused an altered B-cell differentiation and homing to peripheral lymph nodes, but they had normal levels of α_4_ and α_L_ integrins. RIAM deletion in B-cells had no impact on α_4_ and α_L_ expression, but cells showed defective homing and adhesion (46), whereas T-cell specific deletion of RIAM resulted in defective α_E_ expression and increased α_4_ (47), and no changes in surface expression of integrins β_1_, β_2_, β_7_ (48). This is consistent with our results that again show integrin subunit specificity. Ena and VASP double knockout showed a specific decrease in α_4_, β_1_ and β_7_ subunits in T cells with a moderate and possibly compensatory increase in α_L_ (49). hMENA silencing in lung and breast cancer cell lines caused a significant reduction in surface α_3_, α_6_ and β_1_, with diminished *ITGB1* (β_1_ gene) mRNA levels (50). This closely matches our results which show that RIAM, VASP and Vinculin knockouts all caused a specific reduction of *ITGAM* (α_M_ gene) and *ITGAX* (α_X_ gene) mRNA levels. Moreover, hMENA expression was required to maintain normal cytoskeletal organization and G:F-actin ratios (50). This result closely resembles our own, where VASP, RIAM or Vinculin knockout cells all had deficient F-actin content.

Alterations in F-actin content and aberrant cytoskeletal morphologies have been described for RIAM (8, 11, 51) and Vinculin deficient cells (52, 53). Previous results from our group, showed that RIAM deficient cells display decreased F-actin content at the phagocytic cup and that this correlates with deficient VASP pSer^157^ phosphorylation and phagocytic cup recruitment, thereby explaining the deficiency in phagocytosis (17). Much like the results presented here, Ena-VASP-hMena triple knockout cells (mouse fibroblasts and melanoma cell lines) had aberrant lamellipodial morphology which was concordant with a loss of lamellipodial F-actin network organization and decreased F-actin content (54). Our results provide a link between the lack of expression of RIAM, VASP and Vinculin and a reduction in F-actin content, leading to a loss in α_M_ expression which persists under all-*trans* retinoic acid treatment, which is known to induce their expression during neutrophilic differentiation in HL-60 cells. This is supported by the fact that the defect in α_M_ was partially reversed by the actin stabilizing drug jasplakinolide inducing a 2.5-fold increase in expression, indicating that α_M_ expression is heavily regulated by an F-actin dependent mechanism but does not exclude other additional F-actin-independent mechanisms downstream of RIAM, VASP and Vinculin.

In contrast with the results obtained for α_M_, jasplakinolide treatment had a minor effect on α_X_, inducing a non-significant 1.3-fold increase in expression for all cells, suggesting that α_X_ expression is less dependent of F-actin, and that other pathways may be more critical. Interestingly, during neutrophilic differentiation granulocyte colony-stimulating factor (G-CSF) signaling to the MAPK/ERK pathway is required to activate AP-1 (55), which in turn regulates α_X_/CD11c expression (56). We have previously reported that RIAM has an impact on ERK1/2 phosphorylation dynamics (17, 57), which may result in activation of transcription factors like the aforementioned AP-1. Expression and activity of IAC proteins could have an impact on the nuclear translocation of transcription factors downstream of the MAPK/ERK pathway, independently of the SRF/MRTF-A pathway, thereby explaining our results.

We also demonstrate that knockout of RIAM, VASP or Vinculin results in a change in subcellular localization of the SRF coactivator MRTF-A, which is consistent with an increase in inactive cytoplasmic G-actin-bound MRTF-A. hMENA knockouts presented a reduction in SRF activity, an effect comparable to that observed using the SRF inhibitor CCG1423 (50). Similarly, it has been shown that VASP controls SRF activity and co-immunoprecipitates with mDIA1 in mouse fibroblasts (33). Mouse hematopoietic stem cells deficient in mDIA2 presented defective engraftment and migration, reduced F-actin content and inhibited transcription of SRF target genes, which included *FHL-2*, *SRF*, *ITGA2*, *ITGAL*, *ITGAM* and *ITGB2* (58).

There are numerous reports on integrin-mediated functions being inhibited in SRF or MRTF-A deficient cells (35, 36) and that MRTF-A loss-of-function mutations lead to severe immunodeficiency (59). Interestingly a β_2_-Kindlin3-SRF-MRTF-A pathway is proposed to regulate dendritic cell function (34). Our results expand upon this discovery suggesting that disruption of integrin proximal components such as RIAM, VASP or Vinculin can cause severe defects in integrin expression through a dysregulation of the MRTF-A subcellular localization. We also demonstrate that modulation of the G:F-actin ratio using jasplakinolide renormalizes MRTF-A distribution, and that this effect goes in parallel with an increase in α_M_ expression in all knockout cell lines.

We also demonstrated that the Four-and-a-Half LIM domain protein FHL-2, a SRF regulated gene itself, which has been described to compete with the coactivator MRTF-A for SRF binding (37), shifts localization in the absence of RIAM, VASP or Vinculin expression. Whereas FHL-2 is enriched at sub-membranous zones in HL-60 cells, knockouts show an increased nuclear FHL-2 signal, which can be reduced by jasplakinolide treatment. These results suggest that FHL-2 can be sequestered close to integrin adhesion complexes, whereas RIAM, VASP and Vinculin deletion significantly favors its nuclear translocation. In fact, FHL-2 has been described to directly bind several integrin subunits, including β_2_ through its N-terminal LIM domains (60, 61) and is capable of binding α-actin mainly through its C-terminal LIM domains (3 and 4) (62). FHL-2, much like Vinculin and RIAM (63, 64), is described to form part of a mechanosensitive pathway, regulating protein expression according to substrate rigidity and the strength of the adhesion (65). Although, a direct interaction between FHL-2 and VASP, Vinculin or RIAM has not been described yet, the evidence in the literature supports this idea. Both VASP and Vinculin directly interact with the focal adhesion proteins, Zyxin and Paxillin (66–73), and these interactions take place *via* their LIM domains. FHL-2 directly binds to Growth factor receptor-bound protein 7 (Grb7) in a tyrosine phosphorylation-dependent manner *via* its RA and PH domains (74). Since RIAM shares a similar molecular architecture, with an RA-PH module and Proline-rich regions (8–11, 57), and has therefore been compared to the Grb7 protein family (8, 9, 75), it is also possible that RIAM directly interacts with FHL-2. Alternatively, this interaction could also be indirect, through RIAM-mediated recruitment of VASP (17) or Vinculin (63, 76).

Finally, we show that the decrease in α_M_β_2_ and α_X_β_2_ integrin expression observed in RIAM, VASP or Vinculin cell knockouts occurs during all-*trans* retinoic acid induced neutrophilic differentiation of HL-60 cells. A failure to upregulate these integrin subunits might have important significance during the differentiation program of myeloid cells.

Myelopoiesis requires the hierarchical and sequential activation of transcriptional programs controlled mainly by the transcription factors PU.1 and CEBPα (77). PU.1 upregulates α_M_ expression in promyelocytic cell lines in response to all-*trans* retinoic acid (78–80) and this effect has been shown to be in response to G-CSF-Stat3 signaling (81). Vav1 activation through tyrosine phosphorylation helps to drive PU.1 mediated upregulation of α_M_, although the authors found the formation of PU.1 complexes on the *ITGAM* promoter in Vav1 knockouts, suggesting that this may be a feedforward loop (82). Vav1 is upregulated after all-*trans* retinoic acid treatment (83, 84) and its tyrosine phosphorylation is induced downstream of integrin activation (10, 85–89). Therefore, the notion that the expression of RIAM, VASP and Vinculin might help to further drive this purported feedforward loop is given credence.

**Figure 8:**
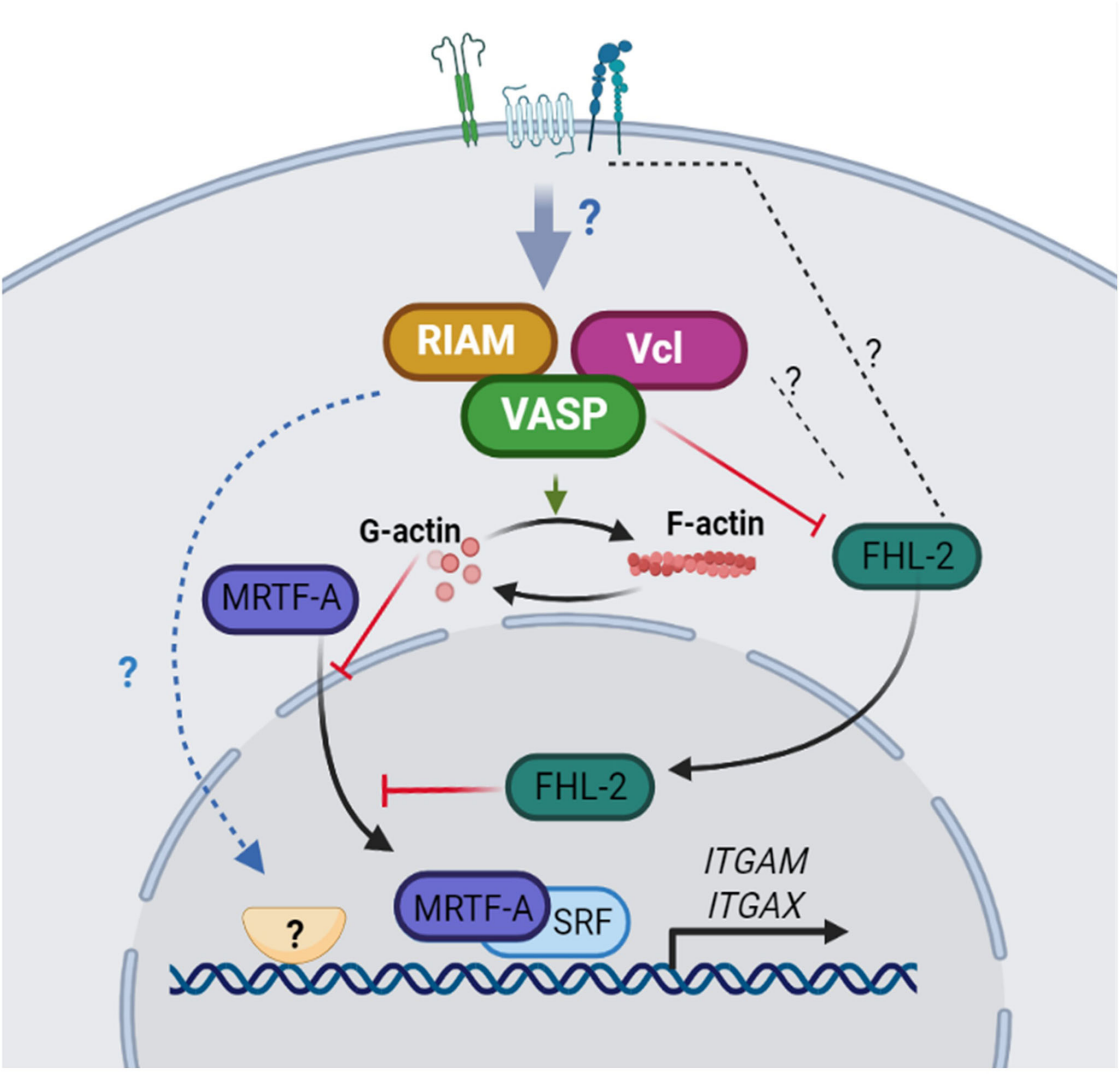
A model for integrin α_M_ and α_X_ upregulation dependent on RIAM, VASP and Vinculin expression. The expression of RIAM, VASP and Vinculin downstream of receptor signaling, contributes to increase the polymerization of actin. The reduced G-actin content allows MRTF-A translocation to the nucleus where, in combination with the SRF transcription factor promotes α_M_ expression predominantly. Simultaneously, FHL-2 is sequestered in the plasma membrane, possibly by interacting with integrins or associated components, reducing FHL-2 co-repressor activity on SRF and allowing integrin expression. In addition, RIAM, VASP and Vinculin could regulate the activity of other potential transcription factors involved in the control α_M_ and α_X_ expression.

Based on this model, an initial transcription of integrin subunits could occur *via* myelopoiesis-specific transcription factor networks like PU.1, C/EBPα, C/EBPϵ and Gfi-1, which are also involved in the expression of G-CSF receptor. Concomitantly, VASP, RIAM and Vinculin could be upregulated, which concurs with our observations noting RIAM and VASP upregulation after all-*trans* retinoic acid treatment and the appearance of an upper band consistent with the phosphorylated form of VASP (**Supplementary Figures 2B, C**) (11). RIAM/VASP/Vinculin activity downstream of different receptors would increase F-actin content allowing MRTF-A/SRFα mediated upregulation of α_M_ with concomitant FHL-2 sequestration at the membrane. Additionally, activation of other transcription factors induced by RIAM/VASP/Vinculin activity may contribute to regulate α_M_ and α_X_ expression (**Figure 8**). Deficiency in α_M_β_2_ and α_X_β_2_ expression has not been described in RIAM, VASP or Vinculin knockout mice (38, 45, 53, 90, 91). This discrepancy could be explained by the existence of compensatory mechanisms operating during myelopoiesis, involving homologous proteins and/or alternative signaling pathways, including those that promote F-actin polymerization. Our results therefore highlight the existence of a pathway that occurs during all-*trans* retinoic acid-induced differentiation of HL-60 cells that works to ensure the upregulation of α_M_β_2_ and α_X_β_2_ integrins and subsequently the acquisition of a phagocytic phenotype.

## Data availability statement

The original contributions presented in the study are included in the article/**Supplementary Material**. Further inquiries can be directed to the corresponding authors.

## Author contributions

AT-G, and EL, contributed to the conception and design of the study. Data acquisition and analysis was conducted by AT-G and EL, with experimental contributions by TF and BC (integrin expression analysis), CG-E and IC (western blot analysis) VT (CRISPR/Cas9 cloning). AT-G, and EL wrote the original draft. Scientific consultation was provided by PR. Final writing and editing were performed by AT-G, CC and EL. All authors contributed to the article and approved the submitted version.

## Funding

This work has been supported by Ministerio Español de Economía y Competitividad (MINECO) grants: SAF2016-77096-R and PID2021-123199OB-I00 (E.L. and C.C.), the CAM (Comunidad Autónoma de Madrid) research agency through grant IND2020/BMD-17364 (P.A.R. and T.F.), and fellowship from MINECO to A. T-G. (FPU15/05349). 

## Conflict of interest

The authors declare that the research was conducted in the absence of any commercial or financial relationships that could be construed as a potential conflict of interest.

## Publisher’s note

All claims expressed in this article are solely those of the authors and do not necessarily represent those of their affiliated organizations, or those of the publisher, the editors and the reviewers. Any product that may be evaluated in this article, or claim that may be made by its manufacturer, is not guaranteed or endorsed by the publisher.
