# Supplementary material for: Expression of the phagocytic receptors αMβ2 and αXβ2 is controlled by RIAM, VASP and Vinculin in neutrophil-differentiated HL-60 cells
Source: Front Immunol. 2022 Sep 27;13:951280. doi: 10.3389/fimmu.2022.951280 (PMC9552961; doi:10.3389/fimmu.2022.951280)
Supplement: Supplementary file 1 [file DataSheet_1.docx]

**Supplementary material**

**
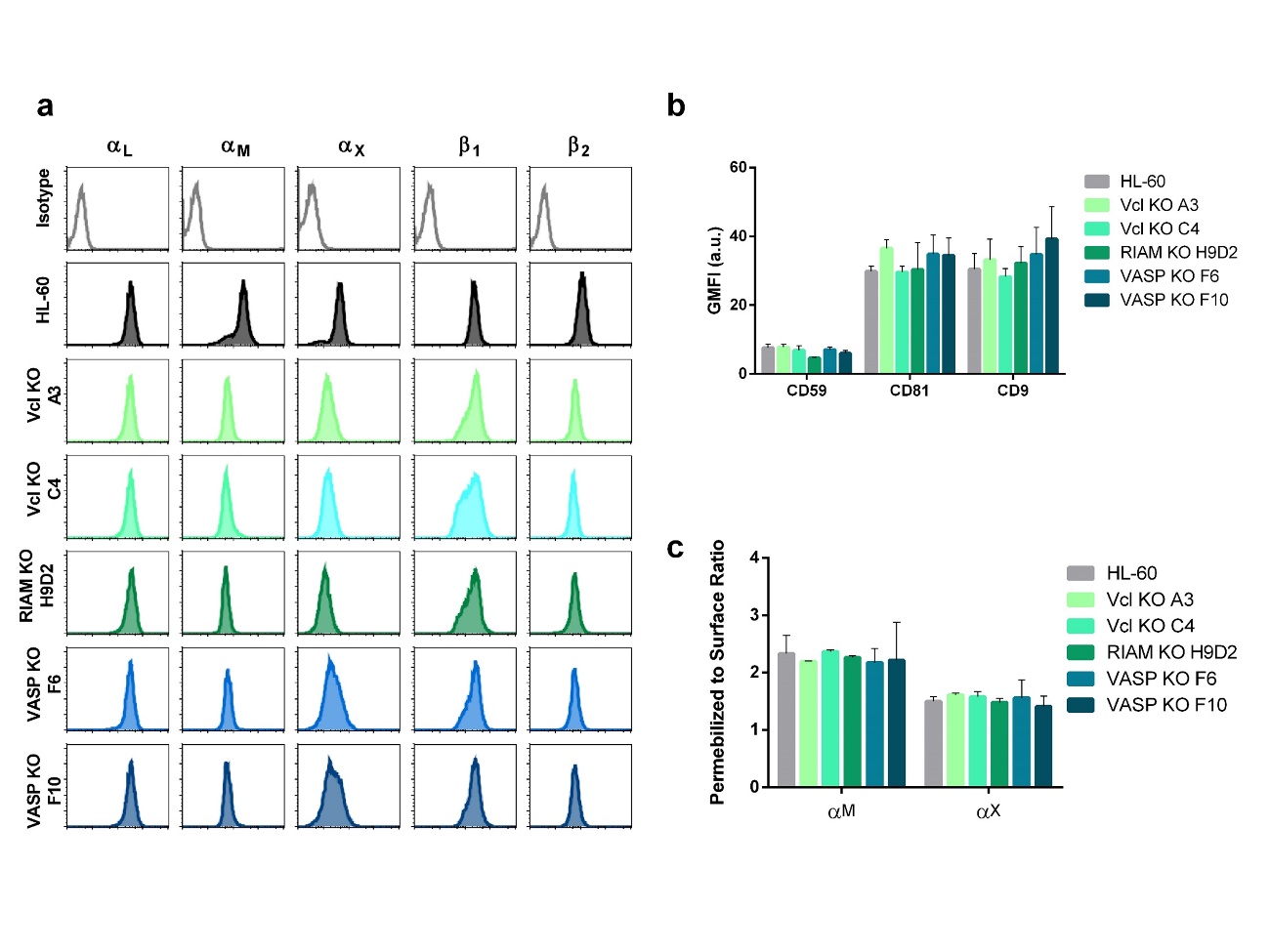
**

**Supplemental Figure 1.**

**a)** Expression of α_M_, α_L,_ α_X,_ β_1_, β_2_ integrin subunits was determined for RA-differentiated HL-60 parental cells and the Vinculin, RIAM and VASP knockout cell lines. Flow cytometry profiles from a representative experiment corresponding to Figure 2a are shown. **b**) Neutrophilic-like Vinculin (Vcl), RIAM and VASP knockout cell lines and HL-60 parental cells were stained with monoclonal antibodies specific for the tetraspanins CD81 and CD9, or cell surface molecule CD59. Geometric Mean Fluorescence Intensity (GMFI) was obtained by flow cytometry. The results show data from 3 independent experiments done in duplicate and represented as mean±SD, where the error bars denote standard deviation. **c)** Neutrophilic-like Vinculin (Vcl), RIAM and VASP knockout cell lines and HL-60 parental cells were permeabilized with 0,1% Triton X-100 for 10 minutes or left untreated. Cells were stained for α_M_ and α_X_ expression and GMFI was obtained by flow cytometry and represented as the ratio of permeabilized staining to surface staining. Data is from 6 independent experiments done in duplicate. Error bars denote standard deviation. Significance (ANOVA test) has been calculated with respect to HL-60 controls, however, no significance was found between samples.


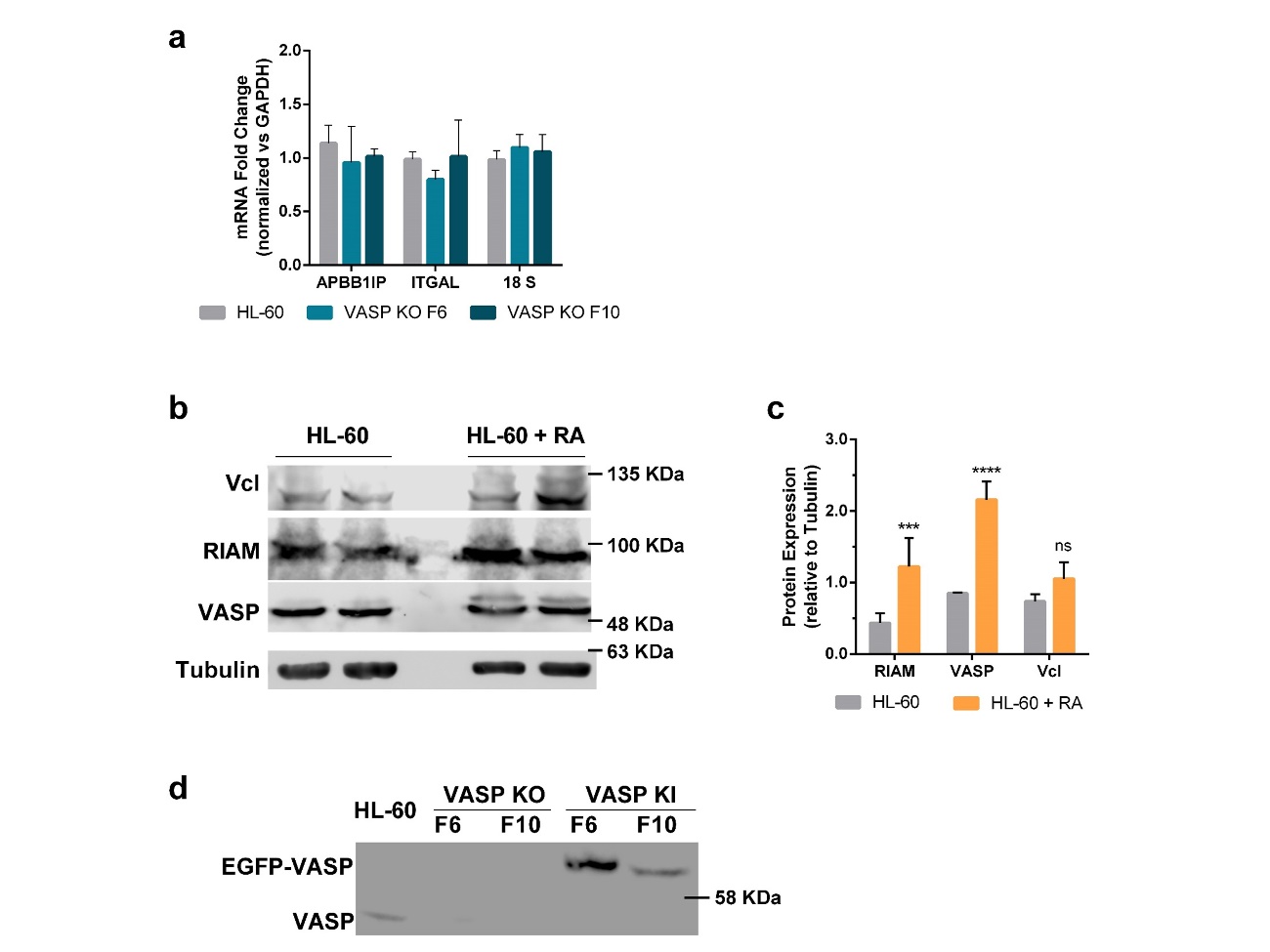


**Supplemental Figure 2.**

**a)** Expression of APBB1IP (RIAM) and ITGAL (integrin α_L_ gene) and 18S rRNA mRNA levels were determined by RT-qPCR in neutrophil-like HL-60 cells and VASP F6 and VASP F10 knockout clones. Results are represented as relative to GAPDH mRNA levels and are from 3 independent experiments done in triplicate. Data are presented as mean±SD, where the error bars denote standard deviation. **b)** and **c)** Cell lysates from undifferentiated HL-60 cells or RA differentiated HL-60 cells (HL-60+RA) were analyzed by western blot for RIAM, VASP and Vinculin expression. The images show representative results from two experiments done in triplicate. Protein band intensity is represented as relative to Tubulin. Significance (ANOVA) has been calculated with respect to HL-60 controls, *** denotes p<0.005, **** p<0.0001, and ns denotes no significance. **d)** VASP KO F6 and F10 clones were transduced with a EGFP-VASP retroviral plasmid and the corresponding polyclonal knock-in cell lines VASP KI F6 and VASP KI F10, were generated. Expression of VASP was analyzed by western blot in HL60, VASP KO F6 and F10 clones and their respective VASP KI F6 and VASP KI F10 polyclonal cell lines.
